# Online-based interventions for sexual health among individuals with cancer: a systematic review

**DOI:** 10.1186/s12913-018-2972-6

**Published:** 2018-03-07

**Authors:** Hee Sun Kang, Hyun-Kyung Kim, Seong Man Park, Jung-Hee Kim

**Affiliations:** 1 Red Cross College of Nursing, Chung-Ang University, 84 heukseok-Ro, Dongjak-gu, Seoul, 06974 Republic of Korea; 2 Department of Nursing, Korean Christian University, 47 Kkachisan-ro 24 gil, Gangseo-gu, Seoul, 17520 Republic of Korea; 3 School of General Education, Dankook University, 119, Dandae-ro, Dongnam-gu, Cheonan-si, Chungnam 31116 Republic of Korea; 4 College of Nursing, The Catholic University of Korea, 222 Banpo-daero, Seocho-gu, Seoul, 06591 Republic of Korea

**Keywords:** Cancer, Online, Psychoeducation, Sexual health

## Abstract

**Background:**

Online interventions have the advantages of being widely available, accessible, comfortable, cost effective, and they can provide tailored information and support. Despite these benefits, the effects of specifically devised online intervention programs for cancer patients’ sexual problems are somewhat unclear. The aim of this review is to describe online-based interventions and to assess their effects on sexual health among cancer survivors and/or their partners.

**Methods:**

We investigated the effects of online sexual interventions among individuals with cancer or their partners. Among these, we considered 4 eligible articles.

**Results:**

Despite the diversity of contents of the interventions, the identified modes of delivery among most of the interventions were as follows: education, interactive methods, cognitive behavior therapy, tailored information, and self-monitoring. Methods of monitoring the interventions, including the utilization of the web site and post-treatment program rating, were reported. All the online intervention programs incorporated a focus on physical, psychological, cognitive, and social aspects of sexual health. Significant effects on patient sexual function and interest and the psychological aspect of sexual problems were reported.

**Conclusion:**

This study provides evidence that online-based interventions would be effective in improving the psycho-sexual problems of cancer survivors and their partners.

## Background

With cancer incidence rates increasing, the time of survival after diagnosis of cancer has also increased, and improvement of the quality of remaining life has gained much attention [1]. Sexuality and intimacy are important features of quality of life that determine fundamental human wellbeing; cancer has long lasting impacts on physical, emotional, psychological, and social aspects of well-being [2].

Cancer patients face sexual difficulties because of chemotherapy, radiotherapy, surgery, and cancer itself [3–5]. Common dysfunctions among female cancer patients include vaginal dryness, dyspareunia, premature menopause, loss of sexual desire, and alterations in body image [6, 7]. Males with cancer may have erection problems, ejaculation dysfunction, and loss of sexual desire during their treatments [8, 9].

Although many sexual problems in cancer patients with chemotherapy have been identified, it is difficult for medical staff to provide appropriate information and support [4, 10, 11]. Despite the needs of survivors and their partners who seek information and support pertaining to treatment, physical changes, and sexual and psychological responses after cancer diagnosis [12], it is not easy for cancer survivors to learn and discuss concerns about sex with medical staff [13]. Unless health care professionals address sexual issues first, cancer patients rarely reveal their sexual concerns [9].

Additionally, health care providers experience obstacles, such as discomfort with discussion and lack of available resources [14, 15]. Initiation of conversations regarding sexual issues tends to be difficult and insufficient [16]. Barriers to addressing patient sexual concerns that have been identified among nurses are discomfort and feeling either embarrassment or a lack of confidence in addressing sexual issues [17, 18]. Health care professionals often avoid or fail to effectively inform and educate patients about sexual changes during and after the treatment [14].

With growing recognition of the internet and communication technology in health care, online-based interventions provide alternatives that can overcome shortages of face–to–face interventions in sexual health areas. Online interventions have the advantages of being widely available and accessible, comfortable, cost effective, and can provide tailored information and support [19]. Internet-based supports ameliorate stress, enhance sense of safety with sensitive issues, provide evidence-based resources about sexual health in cancer [20], and are particularly likely to provide well-matched solutions to inadequate supportive care [21]. Accessing the internet to search for information regarding sexual health is common for young people because of privacy concerns [22]. Furthermore, cancer patients who have difficulty attending outpatient appointments, and those who experience discomfort when discussing sensitive sexual issues, could benefit from online interventions [23]. Internet-based resources may play a role in linking patients with their healthcare professionals and with others in similar circumstances, or in providing educational resources [24].

Several reviews regarding online-based psychoeducational programs dealing with psychosocial or physical aspects of cancer patients have been conducted and have reported positive relationships between the use of online or interactive education programs through the internet and the knowledge levels of breast cancer patients [25]. Additionally, internet-based support programs have had positive effects in relation to alleviating cancer patients’ psychosocial and physical symptoms [26], empowering patients, and encouraging their physical activity [27]. Despite these positive results, the effects of specifically devised online intervention programs for cancer patients’ sexual problems are somewhat unclear.

Therefore, the aim of this review is to describe online-based interventions and to assess their effects on sexual health among cancer survivors and/or their partners.

## Methods

### Data sources and searches

To identify studies pertinent to this review, empirical articles on the effects of online sexual interventions for cancer patients or their partners were investigated using the following search terms: sexual intervention, cancer, oncology, internet, web, and online without a time limit (until December 2015). For this investigation, five online databases, which were CINAHL, Cochrane, EBSCO, PROQUEST, PubMed, and PQDT, were used.

**Fig. 1:**
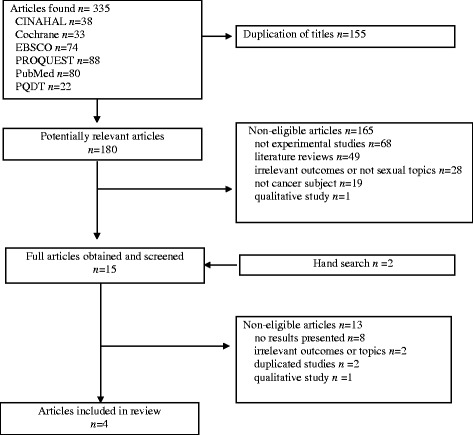
PRISMA Flow Chart

The inclusion criteria for this study included the following elements: (1) use of an online intervention as a major part of the intervention, (2) study of cancer survivors, and (3) international literature written in English. Studies were not included in this study if (1) the focus of the intervention was not on sexuality, (2) they were only a computer assisted assessment study, 3) there was no report of study results on sexual aspects, or (4) they were submitted journal letters and conference proceedings. The initial search yielded 335 articles; then, the number of articles was narrowed down. Through this process, ineligible articles were excluded, resulting in a total of four (Fig. 1).

### Quality appraisal

Two independent reviewers examined the four selected studies for potential bias, prior to inclusion in the review. The critical review checklist for studies, published by Law et al. [28] was adopted. The checklist contains 8 criteria and 15 items for detecting potential bias. Overall, the studies were evaluated as appropriate according to the 15 appraisal items (Table 1). In addition, we reviewed six facets of the guidelines for executing and reporting internet intervention research [29]. All studies reported information about the type and dose of intervention, support (professional or other), program interactivity, multimedia channel of delivery, and degree of synchronicity.

**Table 1:** Methodological Quality of the Studies

Author (Year)	Classen (2013) [20]	Schover (2012) [30]	Schover (2013) [31]	Wootten (2014) [32]
Study Purpose Was the purpose stated clearly?	Y	Y	Y	Y
Literature Was relevant background literature reviewed?	Y	Y	Y	Y
Design	RCT	RCT	RCT	RCT
Sample description Was the sample described in detail?	Y	Y	Y	Y
Sample size justification Was sample size justified?	Y	N	N	N
Reliable measures Were the outcome measures reliable?	Y	Y	Y	Y
	Cronbach’s α were reported	Cronbach’s α were reported	Cronbach’s α were reported	Cronbach’s α were reported
Valid measures Were the outcome measures valid? Validity was reported in a prior study	Y	Y	Y	Y
	FSDS-R: construct validity HADS & IIRS: discriminant validity	IIEF: validated in 32 languages FSFI: reported convergent and discriminant validity BSI-18: incremental validity A-DAS: discriminant validity	FSFI: reported convergent and discriminant MSIQ: construct validity BSI-18: incremental validity QLACS: convergent validity	IIEF: validated in 32 languages DASS-21: convergent validity KMS & DSC: discriminant validity
Intervention description Intervention was described in detail?	Y	Y	Y	Y
Contamination Clients in the control group did not receive treatment inadvertently?	Y	Y	Y	Y
Cointervention Cointervention was avoided?	Y	Y	Y	Y
Reporting results Results were reported in terms of statistical significance?	Y	Y	Y	Y
Analysis methods Were the analysis method(s) appropriate?	Y	Y	Y	Y
Clinical importance Clinical importance was reported?	Y	Y	Y	Y
Drop-outs Drop-outs were reported?	Y	Y	Y	Y
Conclusion and Implications Conclusions were appropriate given study methods and results	Y	Y	Y	Y

*RCT* Randomized Controlled Trial, *Y* Yes, *N* No, *FSDS-R* Female Sexual Distress Scale-Revised, *HADS* Hospital Anxiety and Depression Scale, *IIRS* Illness Intrusiveness Ratings Scale, *IIEF* International Index of Erectile Function, *FSFI* Female Sexual Function Inventory, *BSI-18* Brief Symptom Inventory-18, *A-DAS* Abbreviated form of the Dyadic Adjustment Scale (A-DAS), *MSIQ* Menopausal Sexual Interest Questionnaire, *QLACS* Quality of Life in Adult Cancer Survivors, *DASS-21* Depression anxiety and Stress Scales, *KMS* Kansas Marital Satisfaction Survey, *DSC* Dyadic Sexual Communication

All studies clearly stated their purpose, reviewed relevant background literature and employed randomized controlled trials (RCT). However, one study was a pilot study [20]. The sample size justifications were not reported in three of the four studies. Outcome measures were reliable and valid for all variables. Interventions were described in detail and the authors reported who ran the internet-based interventions and what their roles were, and reported study results clearly. Dropout rates were described and intention-to-treat (ITT) analysis was conducted. Appropriate conclusions were drawn from the study methods and results.

Our methodological quality assessment showed that there was room for improvement regarding obtaining a higher level of quality. In the four studies we reviewed, whether the sample size was properly decided through an adequate power analysis was not clearly addressed. In addition to randomization, all studies tested baseline characteristics. In a study, the groups were very similar in demographic and medical variables and did not differ significantly except the fact that face-to-face group has a shorter duration of follow-up cancer treatment [30]. In a study by Schover et al. [31], the self-help and counseled groups did not differ significantly on any variables. The outcome and demographic variables were not significantly differed, except that Australian born patients were more (85%) in MRA (My Road Ahead)-only group compared to 79% in Forum-only group and 63% in the MRA and Forum group in Wootten et al.’s study [32].

Also, participants who completed the intervention may differ from those who did not, and this might lead to over- or underestimation of intervention effects. However, the studies we reviewed included all eligible participants in the analysis, conducting intention-to-treat (ITT) analyses. This ensured the validity of intervention efficacy, providing unbiased assessments of intervention efficacy.

### Data extraction

Based on the guide to reporting of interventions, the following categories were created. The subjects (country, target population, participation of couple, comparison group, age, and sample size) were included. Information was extracted concerning the description of the intervention (brief name), mode of delivery (interactive and facilitating, cognitive behavioral therapy, self-monitoring, tailored information), and monitoring (intervention provider, follow up period, drop-out rate, participants’ reaction).

The domains of evaluation in each study were divided into sexual, psychological, cognitive, or social aspects. Sexual aspects of online content included sexual changes and activity, sexual function, menopause, fertility, special care for incontinence, and ostomy care in relation to sexual activities. Psychological aspects included emotional aspects of sexuality. Cognitive aspects included content such as body image and self-identity regarding sexuality. Social aspects included content involving relationships and communication.

Since there was considerable diversity, such as differences in intervention characteristics, outcome measures, and cancer types between studies, narrative analysis was performed to identify the effects of the online sexual health interventions.

## Results

### Intervention characteristics of the studies

Characteristics of the reviewed studies are shown in Table 2.

**Table 2:** Intervention Characteristics of the Studies

Categories	Subcategories	Classen (2013) [20]	Schover (2012) [30]	Schover (2013) [31]	Wootten (2014) [32]
Subject	Country	Canada	USA	USA	Australia
	Target population	Gynecologic cancer	Prostate	Breast or gynecological	Prostate
	participation of couple	No	Yes	Yes	Yes
	Comparison Groups	Waitlist group	Waitlist and FF	Self-help group	Receiving forum only
	Mean Age (Trt/Cont Years)	39.9/ 44.6	64.8/ 64.7	52.0/ 54.0	61.0
	Sample Size (Trt/Cont)	13/ 14	25/ 26	27/ 31	33/ 35/ 36
Description of intervention Mode of delivery	Brief name	-	CAREss	Tendrils	My Road Ahead
	Interactive& facilitating	Support group, discussion forum, & chat	Email & phone reminders	–	Forum automated feedback, a log book, & book mark
	CBT	–	C-B homework	C-B exercise	C-B therapy
	Self-monitoring	–	–	–	Weekly mood monitor
	Tailored information	Post message & real time chat	E-mail feedback	Counseling, Video of real experience	Video of real experience
	Intervention period (weeks)	12	12	12	10
	Intervention dose	60 min for chat, 6 topics	8 sections	6 sections	6 modules
Monitoring	Intervention provider	Psychologist Radiation oncologist Forum facilitator	Therapist	Mental health professional	Psychologists
	Password protected	Not reported	Not reported	Yes	Yes
	Follow up period (months)	4, 8	1, 3, 6, 12	3, 6	4, 2.83
Evaluation	Drop-out rate	30.7	33.0	22.0	41.0
	Participant’s reaction	Satisfaction (Exit questionnaire)	Internet usage	Internet usage	Internet usage
	Sexual	Illness intrusiveness (IIRS) (−)	Erectile function (IIEF) (+) Female sexual function FSFI)(+)	Female sexual function (FSFI) (+) Menopausal sexual interest (MSIQ) (+)	Erectile function(IIEF) (−)
	Psychological	Anxiety and depression (HADS)(−) Sexual distress (FSDS-R) (−)	Distress (BSI) (−)	Quality of life (QLACS) (+) Distress (BSI-18 GSI score) (+)	Psychological distress (DASS-21) (+)
	Social	Intimacy (IIRS) (−) Relationship (IIRS) (−)	Dyadic adjustment (A-DAS) (−)		Relationship satisfaction (KMS) (−) Communication within relationships (CPQ-SF) (−) Sexual communication (DSC) (−)

*Trt* Treatment; *Cont* Control, *FF* Face to Face, *C-B* Cognitive Behaviors, *IIRS* Illness Instrusiveness Rating Scale, *HADS* Hospital Anxiety and Depression, *FSDS-R* Female Sexual Distress Scale-Revised, *FSFI* Female Sexual Function Inventory, *BSI* Brief Symptom Inventory, *A-DAS* Dyadic Adjustment Scale, *MSIQ* Menopausal Sexual Interest Questionnaire, *GSI* Global Severity Index, *QLACS* Quality of Life in Adult Cancer Survivors, *DASS-21* Depression Anxiety and Stress Scales, *IIEF* International Index of Erectile Function, *KMS* Kansas Marital Satisfaction Scale, *CPQ-SF* Communication Patterns Questionnaire-Short Form, *DSC* Dyadic sexual communication scale

#### Subjects

Two studies, Schover et al. [31] and Wotten et al. [32], focused on men with prostate cancer and Classen et al. [20] and Schover et al. [30] assessed women with breast or gynecological cancer. One study [20] included only cancer patients; the remaining three studies [30–32] included the partner as intervention participants as well as the patients. The overall median age of the patients was 52.4 years (range = 39.9–64.8 years). The mean ages ranged 39–54 years, which was relatively younger among the participants in the studies for breast or gynecological cancer patients [20, 30].

#### Description of the interventions and modes of delivery

Despite the diversity of contents of the interventions, the identified modes of delivery among most of the interventions were as follows: education, interactive methods, cognitive behavior therapy (CBT), tailored information, and self-monitoring. These delivery modes were used in various combinations and were adjusted to the cancer patients. To begin with, education was identified as the most frequently used mode; all studies used online educational resources. They included information about physical, psychological, cognitive, and social aspects of sexual health using text, video, audio, and graphics. The interactive methods included a facilitator who guided discussion, a forum, and chat moderated by a support group or health professionals. Three studies [20, 30, 32] used a discussion forum or counseling on the web moderated by health professionals. Additionally, interactive or facilitative methods involved using alternative communication routes such as e-mail, phone, and logbooks which allowed participants to track their status and responses to the interactive exercises. The cognitive behavioral delivery mode, consisting of cognitive behavioral exercises and homework, was used. In terms of tailored information, two [31, 32] of the studies included access to videos of interviews with cancer survivors and vignettes on the web describing common sexual problems and coping strategies. One study provided counseling with participants receiving individual feedback from a psychologist and an oncologist.

Classen et al. [20] conducted an intervention using an asynchronous discussion forum along with the provision of psycho-educational materials dealing with the psychosexual changes associated with gynecological cancer. Schover’s study on a program named *CAREss* [30] included only cognitive-behavioral exercises and homework for couples of prostate cancer survivors. Schover et al. [31] also developed the *Tendrils* website that included instructions on using multimedia, and videos of interviews with women cancer survivors illustrating common problems and coping strategies. In addition, the online intervention provided counseling sessions and behavioral homework. *My Road Ahead,* which has educational resources focusing on self-control using self-directed CBT, has been developed [32]. The educational resources also include videos featuring real patients, health professionals’ commentary, and a forum.

Regarding the description of the intervention, three of the four studies [20, 30, 31] implemented the 12 weeks, and one study [32] provided 6 weeks online intervention. The My Road Ahead program [32] is a six module online program consisting of prostate cancer and you, effective communication, physical changes, sexuality and masculinity, sexuality and intimacy, and planning for the future. In the study conducted by Schover et al. [30], 85% of participants on an average were dosed. There was no significant difference in Web site usage in Schover et al.’s study [31]. Those in the self-help group had an average Web site usage of 108.6 min (SD 141.9) compared to 143.4 min (SD 134.8) in the counseled group.

The counseling sessions integrated the intervention on the web; they included email and then phone reminders [30]. Regular automated feedback was also included as the participants progressed through the program [32]. *My Road Ahead* also monitors mood on a weekly basis based on a graph of mood status generated during the intervention period and includes a logbook and a bookmarking capability according to the participants’ personal preferences.

In terms of intervention provider, all the programs were facilitated by a moderator, whose role included moderating a discussion or introducing new topics, providing expert counseling for sexual problems, maintaining the web site, and discussing behavioral homework. Intervention providers were either a psychologist, oncologist, mental health professional, or a therapist. Additionally, a manual for the therapist was provided to guide the content of counseling [31].

#### Monitoring

Methods of monitoring the interventions, including the utilization of the web site and post-treatment program rating, were reported. Three studies [20, 30, 32] electronically monitored utilization of the web site. There was a trend for usage time across the entire study period to be associated with improvement in sexual interest among the gynecological or breast cancer survivors. However, Schoveret al. [30] reported gender differences in the frequency of visits and completion of the web programs for prostate cancer patient couples. Wootten et al. [32] reported that adherence to the program was moderate and a decrease of content completion rates was observed as participants proceeded through the modules.

Two studies evaluated the users’ experiences with the intervention using questionnaires. Classen et al. [20] found that most of the women indicated that the information was feasible to use, easy to read, and had helped increase their knowledge. Additionally, 67% of participants reported that they had a positive experience by participating in the group and they felt comfortable when they shared their experiences. Fifty-seven percent of participants pointed out that they preferred web-based support groups to face-to-face groups in that they felt more comfortable with the former groups. Schover et al. [31] reported that the only significant difference between groups was that counseled women rated the intervention more positively on addressing emotional concerns.

The longest follow up period for the final evaluation conducted post intervention was 12 months [30]. All the studies in this review described dropout rates; these ranged from 22% to 41%.

#### Evaluation

Regarding outcome measures, physical outcomes were the most commonly used outcome measures, such as erectile function, female sexual function, sexual interest, and illness intrusiveness. All studies assessed psychological or sexual distress as psychological measures, as well as quality of life. All studies used validated questionnaires.

The effects of interventions using online differed among the studies. Three studies reported a significantly higher sexual improvement in the online intervention group [20, 30, 31]. Improvements were reduction in sexual distress [20], increase of erectile function [30], female sexual function [30, 31], and menopausal sexual interest [31].

Also, Schover’s study [30] for couples affected by prostate cancer reported positive effects on partners’ sexual function. Regarding psychological outcomes, two studies [31, 32] reported significant changes in psychological distress and quality of life. Schover [31] measured quality of life and distress and found a significant positive effect in the intervention group. The intervention group for prostate cancer also reported decreased psychological distress compared to the control group that received the forum only [32]. Regarding social outcomes including intimacy, relationship quality, dyadic adjustment, and communication, there were no significant group differences. The pilot study addressing gynecological cancer did not report any differences in sexual, psychological, and social outcomes [20].

### Intervention contents of studies

All the online intervention programs incorporated a focus on physical, psychological, cognitive, and social aspects of sexual health. Physical elements of sexual health after cancer included sexual changes, erectile dysfunction, infertility, menopause, resuming sexual activities, incontinence, and stoma care.

Psychological aspects focused on emotional challenges and expression of affection, while cognitive aspects focused on body image or self-identity. Intimate relationships and communication with partners or others were described in the programs as the social element (Table 3). Classen et al. [20] included psychological aspects such as ways to cope with emotional challenges, explore sexuality, manage symptoms, enhance sexuality and intimacy in intimate relationships, and communicate with a partner, along with aspects regarding body image, menopause, and identity.

**Table 3:** Intervention Contents of Studies

Author (Year)	Content (Major theme)
Classen (2013) [20]	Coping with emotional challenges, exploring sexuality, the effect of cancer treatment on one’s sex life, body image, sudden menopause, managing symptoms, identity, enhancing intimacy and sexuality in intimate relationships, and communication with one’s partner
Schover (2012) [30]	Exercises to increase expression of affection, improve sexual communication, increase comfort in initiating sexual activity, and facilitate resuming sex without performance anxiety. Suggestions were provided to treat postmenopausal vaginal atrophy or cope with male urinary incontinence. Treatments for ED
Schover (2013) [31]	The sexual and fertility consequences of their type of cancer and treatment; genital anatomy, including an interactive, vulvar self-portrait with pain and pleasure mapping; sex after menopause; managing vaginal dryness and pain; causes and treatment options for loss of desire or orgasm problems; ways to improve body image; resuming sex comfortably using sensate focus exercises; sexual issues related to ostomies or incontinence; communication with sexual partners and health professionals; dating; lesbian relationships; and sex after childhood and adolescent cancer.
Wootten (2014) [32]	Prostate cancer and you, effective communication, physical change, sexuality and masculinity, sexuality and intimacy, planning for the future

*ED* Erectile Dysfunction

Schover’s study [30] included issues regarding several exercises designed to increase expression of affection, improve sexual communication, increase comfort in starting sexual activity, and facilitate resuming sex without performance anxiety. Specific recommendations were provided for vaginal atrophy or coping with urinary incontinence, as well as information about the efficacy of treatment of erectile dysfunction after prostate cancer. Cognitive reframing was used to identify participants’ negative beliefs about sexuality. Schover et al. [31] included an educational element concerning the sexual and fertility consequences caused by cancer and treatment, as well as about genital anatomy. Additionally, the following topics were described: causes and treatment choices for loss of desire or orgasm, improving body image, using sensate focus exercises, sexual issues related to ostomies or incontinence, and communication with sexual partners. Wootten et al. [32] described their program with the followin2 subtitles: “prostate cancer and you,” “effective communication,” “physical change,” “sexuality and masculinity,” “sexuality and intimacy,” and “planning for the future.”

## Discussion

This review examined studies on online-based interventions for sexual health to assess their effects on cancer patients. Regarding the population in our review, the studies focused on adult patients or partners with prostate cancer, breast cancer, and gynecological cancer. Significant differences may exist in sexual concerns between patients depending on their different cancer types and stages [33]. Furthermore, cancer survivors with other types of cancers may also have sexual difficulties [34–36]. Thus, more studies evaluating the efficacy of such programs are necessary for individuals with different types of cancer and at different cancer stages. Moreover, discussion of sexual activity or sexual issues is culturally sensitive. All four studies were conducted in Western countries (USA, Canada, and Australia), and the applicability of this evidence to other cultures. Studying diverse cultural settings would increase the generalizability of the evidence.

In addition, insufficient research has investigated evidence-based interventions for the young experiencing fertility distress or sexual difficulties due to cancer, although one study has been proposed to test the effectiveness of web-based intervention for adolescents in reducing sexual problems and fertility distress [37]. Younger cancer survivors may also have a greater need for information on fertility preservation options and the impact of treatment on fertility [38]. We should not overlook the need for more age-appropriate web-based interventions addressing the unique medical, psychosexual, and fertility needs of adolescent cancer survivors.

In two of the studies we reviewed, the interventions were couples-based, and cancer survivors and partners’ needs were considered and then addressed. Support from partners [39] is valuable, and partners are encouraged to be fully or partially involved in the interventions for their own benefit and their partner’s sexual health [40]. Tailored management is required because partners may have different educational and support needs throughout the cancer experience [40–42].

The research in our review used various interactive methods such as a facilitator involved in the discussion, a forum, chat moderated by a support group or health professionals, and alternative communication methods including e-mail, phone, logbooks, and bookmarking. This study suggests that professional psychologist or therapist-supported online-based interventions for cancer patients with sexual difficulties could be more effective than the face-to-face interventions in improving sexual health, which would lower the barriers to seeking help for sexual problems among cancer survivors and partners with sexual concerns. Schover [31] concluded that most participants were satisfied with an online-based program. In addition, Classen [20] reported the positive responses for the contents and easiness of usage. Given these findings, online interventions have potential benefit to support patients with sexual problems.

All the studies in this review described dropout rates; these ranged from 22% to 41%, indicating high dropout rates. The attrition rates for online-based lifestyle modification programs ranged from 0 to 52% according to outcome measures, type of participants, and contents [43]. The issue of non-adherence (low completion rates), which may detract from the effectiveness of an intervention, is common in online-based interventions because of their self-guided nature. High rates of attrition and low adherence constitute methodological challenges in Web-based intervention trials [44]. **S**tudies have found that sophisticated navigation and feedback mechanisms are necessary to avoid high dropout rates [31]. Furthermore, it has been reported that several strategies might have a positive influence on adherence, including email reminders [45], individualized feedback**,** offering therapist guidance, and automated text messages [46–48]. It could be critical for health care professionals to check the patients’ progress and participation to increase adherence. In terms of participants’ characteristics, education level, general health, social functioning, and mental health have been reported as risk factors for higher attrition rates [49]. The researchers should consider strategies that could aid in enhancing adherence to minimize attrition when designing internet-based interventions.

This review found that, overall, online-based interventions are effective in improving sexual health in cancer care. Wotten et al. [32] concluded that an online program could be a successful intervention in reducing the psychological and sexual impact after cancer diagnosis. In addition, improvement in sexual function in men and their partners was shown after program completion [30]. Given these findings, online intervention may well be an acceptable tool to support patients with sexual problems. In line with this finding, previous studies showed benefits of web-based educational interventions for increasing knowledge of the disease [24, 50], as well as reducing depression [51], and cancer-related fatigue and anxiety [52]. However, the studies reported in this review did not include a follow-up period longer than 12 months [31, 32]. Longer follow-up periods should be implemented to determine long-term impacts of interventions.

The studies reviewed here varied in educational intervention content, and considered not only physical aspects of sexual function, but also cognitive and psychosocial aspects such as sexual distress or the quality of sexual relationships, as well as the partner’s perspective in couples-based interventions. This indicates that sexual issues must be approached from a holistic perspective [13, 53, 54].

This study provides an important understanding of how cancer survivors and partners use internet-based information and support systems after cancer surgery or treatment. The results of this study could be used to guide the development of internet-based interventions for cancer patients and partners with sexual distress. Support for the development of comprehensive internet-based interventions including sexual health for cancer patients is necessary not only at the organizational level but also the government level. It will also be helpful to include information on internet-based interventions for sexual health in the admission package, information booklet, and hospital websites as a way of disseminating information.

Some limitations of our study should be noted. Although all studies employed RCTs and methodological quality was acceptable, the first concerns the relatively small number of studies evaluated. We found only four studies, the population of which consisted of those with a history of prostate, breast, or gynecological cancer. We included different cancer types and interventions for couples and individuals as well, although there may be significant differences in sexual concerns between cancer survivors and partners with different cancer types. The variation in the effects of online-based interventions on different cancer types also requires greater clarification to increase its generalizability. Since there are few studies and substantial variability exists across studies, careful attention is required to interpret and generalize the results. Another limitation is that our literature search only included articles in English. There may also be studies published in other languages, as well as unpublished thesis or conference proceedings that we missed.

## Conclusions

Few studies have focused on online-based interventions for sexual health. In general, overall positive effects of online-based interventions targeting sexual health of cancer survivors and their partners were found in this review. Our findings indicate that online-based educational interventions for sexual healthcare feasible and have potential benefits for cancer patients with sexual concerns. The results of these studies suggest that online interventions would be effective in reducing sexual difficulties. However, it should be noted that most included studies were performed with patients with prostate, breast, and gynecological cancer in high-income countries with good web-based technology systems. Therefore, caution should be noted in generalizing from these results.

This review has revealed that more research of high methodological quality with large and various clinical and cultural populations is required to support the positive benefits of online-based intervention for cancer survivors with sexual difficulties. Evaluation of the cost-effectiveness of internet-based interventions focused on sexual problems would be beneficial.

In conclusion, online-based interventions are effective in improving the psycho-sexual functioning cancer survivors and their partners, as well as lowering their barriers to seeking help for sexual and psychological problems. In addition, internet-based interventions may be integrated into other interventions aimed at promoting quality of life and provided to those who are on a waitlist in need of face-to-face interventions.

## Abbreviations

CBT: Cognitive behavior therapy
ITT: Intention-to-treat
RCT: Randomized controlled trials
